# Phenolic Profile and Antioxidant Activity of the Edible Tree Peony Flower and Underlying Mechanisms of Preventive Effect on H_2_O_2_-Induced Oxidative Damage in Caco-2 Cells

**DOI:** 10.3390/foods8100471

**Published:** 2019-10-10

**Authors:** Jinle Xiang, Chengbo Yang, Trust Beta, Shangxi Liu, Runqiang Yang

**Affiliations:** 1 College of Food & Bioengineering, Henan University of Science & Technology, Luoyang 471023, China; Xjl5013@haust.edu.cn; 2 Department of Food & Human Nutritional Sciences, University of Manitoba, Winnipeg, MB R3T 2N2, Canada; Trust.Beta@umanitoba.ca; 3 Department of Animal Science, University of Manitoba, Winnipeg, MB R3T 2N2, Canada; chengbo.yang@umanitoba.ca (C.Y.); Shangxi.Liu@umanitoba.ca (S.L.); 4 College of Food Science & Technology, Nanjing Agricultural University, Nanjing 210095, China

**Keywords:** tree peony flower, HPLC-Q-TOF-MS/MS, phenolic profiles, in vitro antioxidant activity, cellular antioxidant properties

## Abstract

The entire phenolic profiles and antioxidant activities of different organs of the edible tree peony flowers (*Fengdan Bai* (FDB)) were analyzed. HPLC-quadrupole time-of-flight mass spectrometer (Q-TOF-MS/MS) analyses of individual phenolic compounds revealed that the petal and stamen contained higher levels of flavonoid glycosides than other organs (*p* < 0.05). Kaempferol-3,7-di-*O*-glucoside was the dominant flavonoid in these two organs, however, the calyx and ovary contained higher contents of gallic acid derivatives than other organs (*p* < 0.05). Hexa-*O*-galloyl-glucose was the dominant species in the calyx and ovary. At the same concentration of total phenolic extract (TPE), the stamen had the highest protection effect on Caco-2 cell oxidative damage induced by H_2_O_2_. The antioxidant effect was attributed to potent antioxidant capability; restored redox state due to the increased expression of glutathione peroxidase (GSH-Px) and superoxide dismutase (SOD); and improved barrier function of Caco-2 cell owing to increased zonula occludens-1 (ZO-1), CLDN3 (Claudin 3), and occludin mRNA expression. As a new resource food, the edible tree peony flower is a potential functional food material and natural antioxidants resource.

## 1. Introduction

With increasing evidence that reactive oxygen species (ROS) induce oxidative stress during the development of various chronic and degenerative diseases, such as cancer, cardiovascular disease, diabetes mellitus, and aging, radical-scavenging antioxidants for preventing oxidative stress-related diseases have received great attention [1]. The increased demand for new, natural antioxidant foods has generated an interest in edible flowers in recent years [2,3,4].

Tree peony (*Paeonia suffruticosa Andr.*) is native to China with more than 1600 years’ history of cultivation, and is considered as a traditional ornamental and medicinal plant [5]. The flower color of tree peony is diverse with nine categories that include red, white, green, pink, blue, purple, black, yellow, and dual colors [5]. The antioxidant activity is attributed mainly to anthocyanins in the purple, pink, and red categories [6,7], and flavonoids in the yellow and white categories [6,8]. In China, the flowers of tree peony are used as herbal medicines for the treatment of diseases mainly related to irregular menstruation and dysmenorrhea in women [9]. Some of the flowers have also been used to make a variety of delicious folk foods since the Song dynasty (A.D. 960–1279), such as teas, cakes, casseroles, and drinks, owing to the presence of nutrients including proteins, microelements, and vitamins [6]. However, until now, only the flower of one white category, named *Fengdan Bai* (FDB), was officially approved as a new food material by China’s Ministry of Health in 2013. Following the approval, different foods made from the petals, stamens, or the whole flower of FDB have become popular for their flavor and health function. In order to expand the utilization of the approved edible flowers as functional food material, two polysaccharides were purified from the petals of FDB and characterized in our previous study [10]. 

Various phytochemicals, including phenolics [11,12], stilbenes [13], and terpenoids [14,15,16], were identified in tree peony in the previous studies. The phytochemical species and contents in tree peony differ according to the cultivars and organs involved [6,17,18,19]. Tree peony seeds exhibit strong antioxidant activity and phenolic compounds have been verified as the most important antioxidants [11,17,19]. However, the phenolic profiles of the edible tree peony flower FDB have not been identified and the antioxidant properties have not yet been characterized. In addition to free radical scavenging capacity, cellular oxidative models have been widely used to investigate the potential mechanisms of phytochemicals involved in human health [20]. In the present study, following extraction and identification of phenolics in the petal, stamen, calyx, and ovary of the tree peony flower (cultivar FDB), the 2,2′-diphenyl-1-picrylhydrazyl (DPPH), 2,2′-azino-bis (3-ethylbenzothiazoline-6-sulfonic acid) diammonium salt (ABTS^•+^) radical scavenging activities, and oxygen radical absorbance capacity (ORAC) were determined. Furthermore, an oxidative damage model of Caco-2 cells was established by hydrogen peroxide (H_2_O_2_) treatment, and the mechanisms of the extracts on the recovery of the Caco-2 cells from oxidative damage were revealed via examining the antioxidant system and the tight junction protein expression.

## 2. Materials and Methods

### 2.1. Tree Peony Flower Samples

The edible tree peony flowers (*Fengdan Bai*) were collected in full blossom from the campus of Henan University of Science & Technology, China in mid April 2018. The whole flower was freeze-dried and different organs, including petal, stamen, calyx, and ovary, were separated and milled to powder in an electric mill (Micro-Mill, Bel-Art Products Co., Wayne, NJ, USA) with a 60 mesh screen. The ground samples were stored at −20 °C before extraction.

### 2.2. Chemicals

Folin-Ciocalteu reagent, 2,2′-azobis-(2-methylpropionamidine) dihydrochloride (AAPH), 2,2′-diphenyl-1-picrylhydrazyl (DPPH), 2,2′-azino-bis (3-ethylbenzothiazoline-6-sulfonic acid) diammonium salt (ABTS), 6-hydroxy-2,5,7,8-tetramethylchromane-2-carboxylic acid (Trolox), fluorescein, HPLC grade methanol, and MS grade methanol were all purchased from Sigma-Aldrich Chemical Co. (St. Louis, MO, USA). Standards of gallic acid, methyl gallate, rutin, and quercetin were also purchased from Sigma-Aldrich Chemical Co. (St. Louis, MO, USA). Hexanes, sodium hydroxide, hydrochloric acid, and sodium carbonate were purchased from Fisher Scientific (Ottawa, ON, Canada). Hydrogen peroxide (H_2_O_2_) and 2′,7′-dichlorofluorescein diacetate (DCFH-DA) were purchased from Sigma-Aldrich (Oakville, ON, Canada). Hank’s balanced salt solution (HBSS), dulbecco’s modified eagle medium /F12 (DMEM/F12), anti-anti and fetal bovine serum (FBS) T-75 culture flasks, Transwell Permeable supports (0.6 cm^2^, 0.4 μm pore size), and cell culture plate were purchased from Invitrogen, Fisher Scientific (Ottawa, ON, Canada). 

### 2.3. Sample Preparation

The extraction of phenolic compounds was conducted according to the method reported earlier [21] with some modifications. The different organ parts of FDB were defatted twice with hexanes and then extracted three times with 80% methanol for 1 h at room temperature. The supernatants were combined and dried under vacuum at 38 °C, and reconstituted in 50% methanol for use as crude extracts.

### 2.4. Determination of Total Phenolic Content and Total Flavonoid Content

Determination of total phenolic content (TPC) was based on the method described by Xiang, et al. [22] using a 96-well ELX800 microplate reader (BioTek Instruments Inc., Winooski, VT, USA) and gallic acid as the standard. TPC was expressed as milligrams of gallic acid equivalents per gram (g) of the sample (mg GAE/g). Total flavonoid content (TFC) was determined as described by Biney and Beta [23] using rutin as the standard. The results were expressed as milligrams of rutin equivalents per gram (g) of the sample (mg RE/g).

### 2.5. Determination of Antioxidant Capacity In Vitro

DPPH and ABTS^•+^ radical scavenging activities of the four parts of the flower were measured as described by Xiang, et al. [24] using a 96-well ELX800 microplate reader (BioTek Instruments Inc., Winooski, VT, USA). Oxygen radical absorbance capacity (ORAC) was measured as described by Qiu, et al. [25]. The results were expressed as micromole Trolox equivalents per gram of the sample (μmol TE/g).

### 2.6. Identification and Quantification of Phenolic Compounds by HPLC- Quadrupole Time-of-Flight Mass Spectrometer (Q-TOF-MS^2^)

An HPLC (Waters 2695) equipped with a photodiode array (PDA) detector (Waters 996) and autosampler (717 plus, Waters) coupled to a quadrupole time-of-flight mass spectrometer (Q-TOF-MS) (Micromass, Waters Corp., Milford, MA, USA) was employed for LC and mass spectrometric analyses (LC–MS). Samples were eluted through a 150 mm × 4.6 mm, Gemini 5 μm C_18_ 110A column (Phenomenex, Torrance, CA, USA) with a binary mobile phase consisting of A (water with 0.1% formic acid) and B (methanol with 0.1% formic acid). Separation of phenolic compounds was achieved by our previously reported method [26]. The elution procedure was as follows: 0 min 4% B, 18 min 18% B, 35 min 30% B, 58 min 42% B, and 70 min 60% B. Compounds were identified by comparing retention time (RT) and UV spectra information and confirmed by Q-TOF-MS/MS. 

The Q-TOF-MS was calibrated for the negative mode through the mass range of 100–1500 with the resolution of 5000, according to the method previously reported [26]. Full mass spectra were recorded using a capillary voltage of 1.45 kV and a cone voltage of 30 V. The flow rates of cone gas (He) and desolvation gas (N_2_) were 45 and 900 L/h, respectively. The desolvation gas temperature and ion source temperature were set at 250 °C and 120 °C, respectively, while the collision energies of 20, 30, and 45 V were used to acquire the MS^2^ spectra. 

The individual phenolic compounds were separated under the same HPLC conditions described above, and quantification was based on the area of the peak at wavelengths of 280 nm for gallic acid and methyl gallate derivatives, and 350 nm for flavonoid glycosides. The compounds without authentic standards were measured semi-quantitatively according to the method previously reported [8]. Gallic acid was used for quantification of gallic acid, galloyl hexose, and gallotannins, and the contents of individual gallic acid derivatives were expressed as mg GAE/100 g. Methyl gallate (MGA) was used for quantification of methyl gallate and methyl digallates, and the contents of individual methyl gallate derivatives were expressed as mg methyl gallate equivalents per 100 g of the sample. Quercetin was used for quantification of all the flavonoid glycosides, and the individual flavonoid glycosides were expressed as mg quercetin equivalents per 100 g of the sample.

### 2.7. Cell Culture

The non-transformed neonatal human colon cancer cells (Caco-2) were supplied by the American Type Culture Collection (ATCC, Manassas, VI, USA). Cell culture was conducted according the previous study [27]. Culture medium was replaced every 2−3 d, and then treated with trypsin (Fisher Scientific, Ottawa, ON, Canada) and seeded into a 96-well, 12-well, or 24-well plate. Cells were treated with H_2_O_2_ and total phenolic extract (TPE) after six days (100% confluent). 

Oxidative damage was induced by H_2_O_2_. For TPE treatment, a stock solution containing 20 mg/mL of phenolic compound in dimethyl sulfoxide (DMSO, 0.1%) was freshly prepared and diluted in the culture medium. To eliminate the influence of DMSO, equal levels of DMSO were added to all of the experimental groups. The treatments were as follows: (A) prevention: cells were treated with TPE for 1 h, and then 0.8 and 2.0 mM H_2_O_2_ were added for 1 h treatment, respectively. (B) Protection: cells were treated with TPE for 1 h, and then TPE plus 0.8 and 2.0 mM H_2_O_2_ were added for 1 h treatment, respectively. (C) Remedy: cells were treated with 0.8 and 2.0 mM H_2_O_2_ 1 h, and then treated with TPE containing 0.5, 1.0, 10, 50, and 100 μg/mL of the phenolic compound for 4 h.

### 2.8. Cell Viability Assay

Cell viability was measured according to Yang et al. [27]. Briefly, Caco-2 cells were seeded into 96-well plates at a density of 2 × 10^4^/mL and cultured in the culture medium for 6 d (100% confluent). After different treatments described above, the cells were washed once with phosphate belanced solution (PBS). A 100 μL fresh medium containing 10% WTS-1 was added and the cells were incubated for 1.5 h. The absorbance at 450 nm was measured using a Synergy™ H4 Hybrid Multi-Mode Microplate Reader (BioTek, Kobashigawa LC, Tokyo, Japan). Cell viability was presented as a percentage of untreated, control cells.

### 2.9. Transepithelial Electrical Resistant (TEER) Measurement

TEER measurements were performed using the method of Yang et al. [27].

### 2.10. Reactive Oxygen Species (ROS) Assay

Cells were cultured onto coverslips (24-well plate, Fisher Scientific) for two days and then treated with 2 mM of H_2_O_2_ for 1 h, and followed by TPE containing 1.0 μg/mL of the phenolic compound from stamen for 4 h (0.5 mL). At the end of treatment, 0.5 mL DCFH-DA solution (10 µM in medium) was added and cells were incubated for 30 min at 37 °C continuously. Cells were then fixed using 4% paraformaldehyde (PFA) for 15 min at room temperature. Cells were then washed twice with PBS and mounted using Vectashield Mounting Medium with 4′,6-diamidino-2-phenylindole (DAPI, Vector Laboratories, Inc. Burlingame, CA, USA). The images were taken by a Zeiss Fluorescence Microscope (Carl-Zeiss Ltd., Toronto, ON, Canada). 

### 2.11. Glutathione (GSH) Content Determination

Total GSH and glutathiol (GSSG) contents were determined using a Glutathione Colorimetric Detection Kit (Catalog Number: EIAGSHC, Invitrogen, Carlsbad, CA, USA) according to the instructions. Cells were cultured in 12-well plates and treated with 2 mM of H_2_O_2_ for 1 h and then treated with 1.0 μg/mL of TPE from stamen.

### 2.12. RNA Extraction and Real-Time PCR

After being treated with 2 mM of H_2_O_2_ for 1 h and then treated with 1 μg/mL of TPC from the stamen, cells were washed with PBS once. Total RNA was extracted from Caco-2 cells using Trizol reagent (Invitrogen, Fisher Scientific; Ottawa, ON, Canada) following the manufacturer’s protocol. The, 1 μg of total RNA was reverse transcribed into cDNA using the iScriptTM cDNA Synthesis kit (Bio-Rad, Laboratories Ltd., Montreal, QC, Canada) following the manufacturer’s instruction. Quantitative RT-PCR was performed using SYBR Green Supermix (Bio-Rad) on a CFX Connect™ Real-Time PCR Detection System (Bio-Rad, Laboratories Ltd.). The reference gene was β-actin. The primers for real-time PCR analysis were designed with Primer-Blast based on the published cDNA sequence in the GenBank. The detected genes and sequences of primers are listed in Table S1. 

### 2.13. Immunofluorescent Staining

Immunofluorescent staining assay was conducted according to Yang et al. [27] with minor modification. Cells were cultured onto coverslips (24-well plate, Fisher Scientific) for four days and treated with 2 mM of H_2_O_2_ for 1 h, and then treated with 1.0 μg/mL of TPC from stamen. 

### 2.14. Statistical Analysis

The data were analyzed using GraphPad Prism 7.0 (San Diego, CA, USA). The differences of mean values among treatments were determined using one-way analysis of variance (ANOVA) followed by Tukey’s honestly significant differences (HSD) test at *p* < 0.05 significance level. The results are presented as mean ± standard deviation (SD).

## 3. Results 

### 3.1. Identification of Phenolic Compounds by HPLC-DAD-ESI-MS^2^

**Figure 1:**
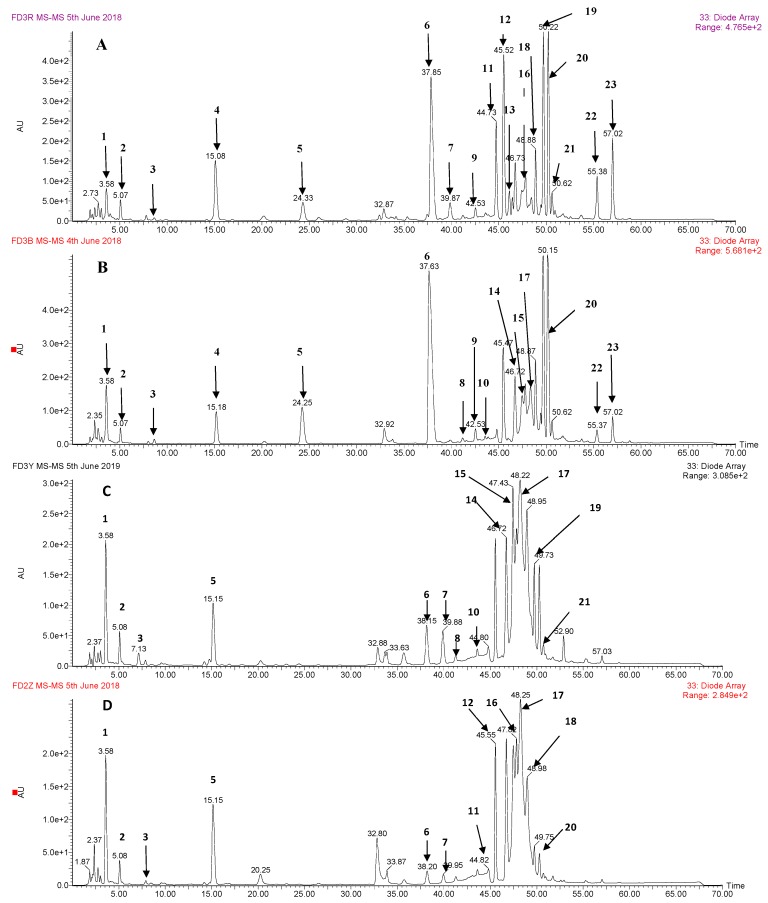
HPLC chromatogram of phenolics from petal (**A**), stamen (**B**), calyx (**C**), and ovary (**D**) of the tree peony flower. AU: absorption unit; e+1: 10 ^1^; e+2: 10 ^2^; number 1, 2, 3, 4, …, 20, 21 corresponds to No. 1, 2, 3, 4, …, 20, 21 listed in Table 1, and 22, 23 was the unidentified compounds.

The representative profiles of the extracts from the different organs of the tree peony flower are shown in Figure 1. The mass and UV spectral characterization for compounds in the representative chromatograms are listed in Table 1, as well as their identification. Twenty-one phenolic compounds were identified in the crude extracts. As shown in Table 1, among these 21 compounds, there were 8 phenolic acids (compound 1–4 and 8–11), 5 gallotannins (compound 12 and 14–17), and 8 flavonoids (compound 5–7, 13, and 18–21). Compounds were identified by comparing with the authentic standards, or tentatively identified by comparing their mass and UV spectral characteristics with those reported in the literature (Supplementary File).

### 3.2. TPC, TFC, and Individual Phenolic Compounds Content

**Figure 2:**
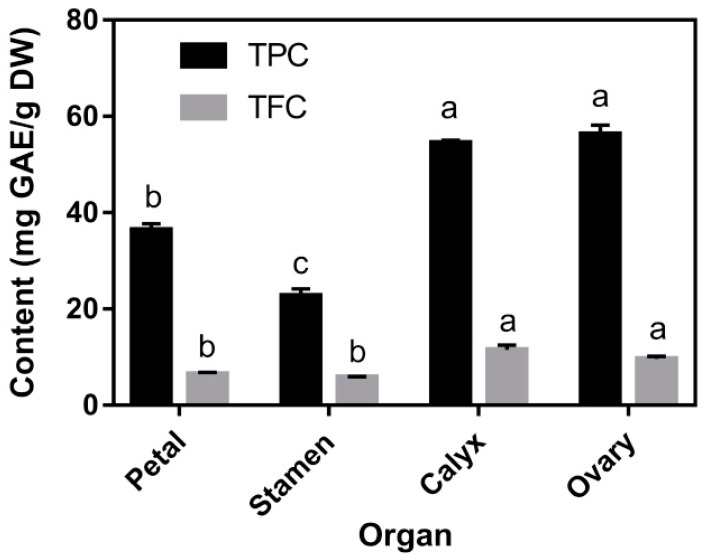
The total phenolic content (TPC) and total flavonoid content (TFC) in the methanol extracts of different organs from the tree peony flower. DW, dry weight of the sample. Results are expressed as mean ± SD. Lowercase letters indicate the significant difference (*p* < 0.05) for TPC and TFC, respectively, among different organs.

TPCs and TFCs of the four organs are shown in Figure 2. The TPC and TFC ranged from 22.76 to 56.29 mg GAE/g DW (dry weight of sample) and 5.81 to 11.35 mg RE/g DW, respectively. The ovary and calyx of the tree peony flower showed significantly (*p* < 0.05) higher TPC than the petal and stamen. The calyx contained the highest TFC (11.35 ± 0.98 mg RE/g DW), while the stamen had the lowest TPC. Generally, petals and stamens of edible flowers are more attractive and are used as ingredients for drinks and other food. From our results, the often overlooked organs of the edible flower, such as the ovary and calyx, may provide more phenolic compounds than other organs. As for the individual polyphenols determined by HPLC analysis (Table 2), the petal and stamen contained higher contents of flavonoid glycosides than other organs. Kaempferol-3,7-di-*O*-glucoside was the major flavonoid in the petal and stamen with levels of 1124.33 and 601.44 mg/100g DW, respectively. Besides kaempferol-3,7-di-*O*-glucoside, apigenin-7-*O*-glucoside and apigenin-7-*O*- neohesperidoside also contributed to the majority of flavonoids in each organ of the tree peony flower. However, compared with the petal and stamen, the calyx and ovary contained higher contents of gallic acid derivatives with a relatively lower content of the identified flavonoid glycosides. Hexa-*O*-galloyl-glucose was the major phenolic in the calyx and ovary with 308.29 and 329.73 mg/100g DW, respectively. In addition, the other two hexa-*O*-galloyl-glucose isomers, together with penta-*O*-galloyl-glucose and hepta-*O*-galloyl-glucose, were also the main phenolic acid derivatives in each organ of the tree peony flower.

### 3.3. Antioxidant Properties In Vitro

To distinguish the antioxidant properties, ORAC, DPPH, and ABTS^•+^ radical scavenging activity assays were applied. As seen in Table 3, the DPPH and ABTS^•+^ radical scavenging activities for the different organs ranged from 207.80 to 444.58 and from 234.58 to 610.21 μmol TE/g, respectively. The methanol extracts of ovary and stamen exhibited the highest and lowest DPPH and ABTS^+^ radical scavenging activity, respectively, which could be attributed to their TPC and TFC. ORAC values of the different organs ranged from 555.11 to 1061.03 μM TE/g, as shown in Table 3. Compared with DPPH and ABTS^•+^, ORAC values of the petal were significantly (*p* < 0.05) higher than that of the other organs. Contrary to DPPH and ABTS^•+^ values, the ovary presented the lowest ORAC values. 

### 3.4. The Protective Effects of TPE against Oxidative Damage Induced by H_2_O_2_ on Caco-2 Cells

**Figure 3:**
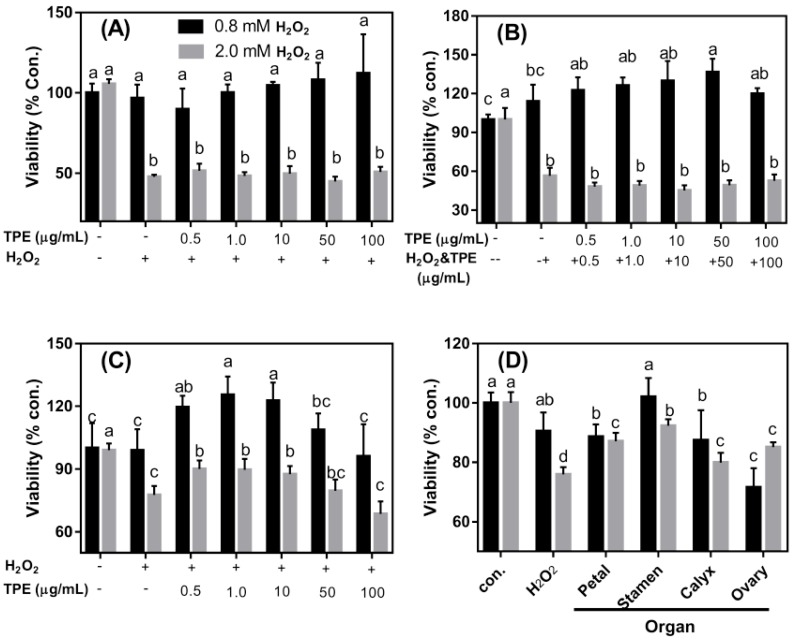
The preventive effect of total phenolic extract (TPE) on Caco-2 cell viability decrease induced by H_2_O_2_. (**A**) Cells were treated with different TPE concentrations for 1 h, and then 0.8 or 2.0 mM H_2_O_2_ was added for a treatment lasting 1 h, respectively. (**B**) Cells were treated with TPE for 1 h and then different concentrations of TPE plus 0.8 or 2.0 mM H_2_O_2_ were added for a treatment lasting 1 h, respectively. (**C**) Cells were treated for 1 h with 0.8 or 2.0 mM H_2_O_2_, followed by treatment with different TPE concentrations lasting for 4 h. (**D**) Cells were treated for 1 h with 0.8 or 2.0 mM H_2_O_2_, and then treated for 4 h with 1 µg/mL TPE from different organs. For H_2_O_2_ treatment, cells were first treated with 0.8 or 2.0 mM H_2_O_2_ for 1 h, and then treated for 4 h with a regular medium. The control was not treated with TPE or H_2_O_2_ containing 0.1% dimethyl sulfoxide (DMSO). The TPE used in Figure A, B, and C was extracted from the whole flower using 80% methanol. The TPE in Figure D was extracted from four different organs. Data are expressed as mean ± SD. The con. means negtive control (no H_2_O_2_ or TPE treatment). Different lower case letters indicate significant difference at *p* < 0.05 for each treatment.

Concentrations of 0.8 and 2.0 mM of H_2_O_2_ were used to induce oxidative damage to Caco-2 cells. Pre-treatment with TPE exhibited no effect on the viability of the cells (Figure 3A). A high concentration of H_2_O_2_ (2.0 mM), pretreatment with TPE for 1 h, followed by TPE plus H_2_O_2_ treatment showed no influence on cell viability (Figure 3B). However, pretreatment with H_2_O_2_ for 1 h followed by treatment with TPE for 4 h significantly improved cell viability (Figure 3C) (*p* < 0.05). The effects of TPE from different organs on cell activity under H_2_O_2_ treatment are shown in Figure 3D. At low concentration (0.8 mM), TPE from various organs did not enhance cell viability (*p* > 0.05). However, the phenolic extract from the ovary significantly reduced cell activity. TPE from the stamen showed no significant effect on cell viability compared with the control and H_2_O_2_ treatment alone. At a high concentration of H_2_O_2_ (2.0 mM), TPE from all organs significantly enhanced cell activity, with the stamen producing the greatest effect (Figure 3D). 

### 3.5. Effect of TPE from Stamen on Redox State of Caco-2 Cell under H_2_O_2_ Treatment

**Figure 4:**
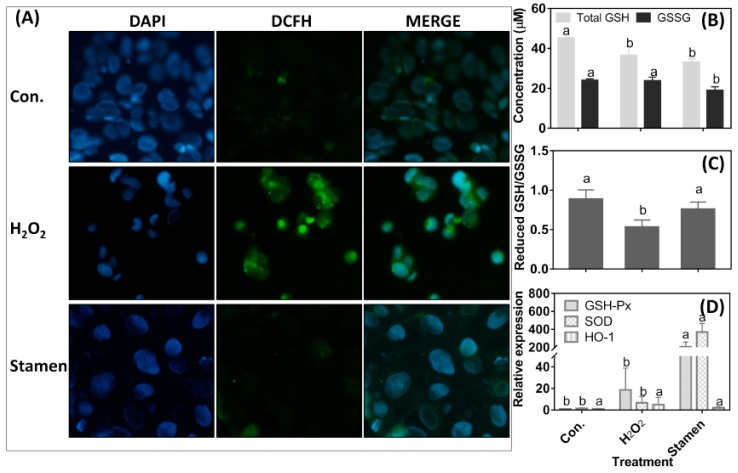
The effect of total phenolic extract (TPE) from stamen on reactive oxygen species (ROS) production (**A**), glutathione (GSH) content (**B**), the ratio of reduced GSH/GSSG (glutathiol) (**C**), and antioxidative enzymes expression (**D**) of Caco-2 cell under H_2_O_2_ treatment. Cells were treated for 1 h with 2.0 mM H_2_O_2_, and then treated for 4 h with 1 µg/mL TPE from the stamen. For H_2_O_2_ treatment, cells were first treated with 2.0 mM H_2_O_2_ for 1 h and then treated with a regular medium for 4 h. DCFH: 2′,7′-Dichlorofluorescin, DAPI: 4′,6-diamidino-2-phenylindole. The con. means negtive control (no H_2_O_2_ or TPE treatment). The magnification in Figure (**A**) is 40×. Data are expressed as mean ± SD. Different lower case letters indicate the significant differences at *p* < 0.05 for each parameter.

Compared with the control, H_2_O_2_ treatment significantly increased the intracellular ROS level. However, the stamen-derived TPE significantly reduced ROS levels generated after treatment with 2.0 mM of H_2_O_2_ for 1 h (Figure 4A). The H_2_O_2_ treatment significantly reduced the total GSH content and, at the same time (*p* < 0.05), it did not alter the GSSG content in the cells. The TPE intervention that followed H_2_O_2_ treatment did not increase the total GSH content, but it significantly decreased GSSG content (Figure 4B). Therefore, the ratio of reduced GSH to GSSG was increased (Figure 4C). As shown in Figure 4D, H_2_O_2_ treatment did not affect the mRNA levels of GSH-Px, SOD, and HO-1. However, the TPE intervention significantly increased the expression of GSH-Px by 204.7 and SOD by 369.2-fold, respectively, when compared with the control (*p* < 0.05).

### 3.6. Effect of TPE from Stamen on Barrier Function of Caco-2 Cell under H_2_O_2_ Treatment

**Figure 5:**
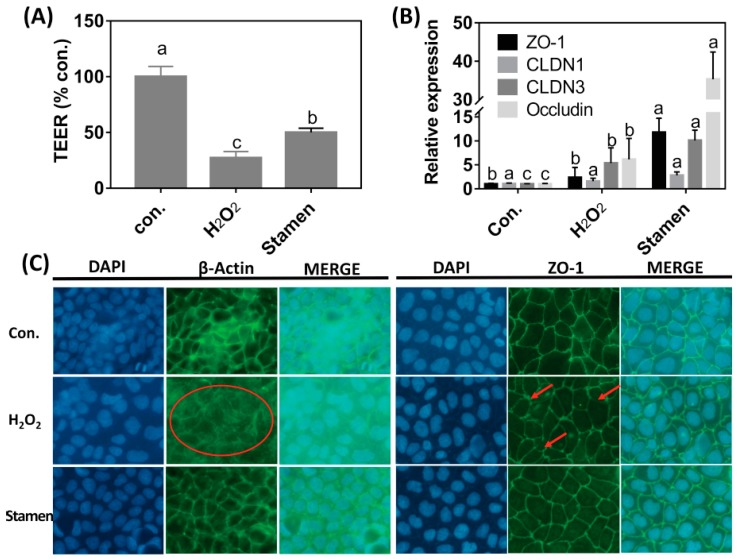
Effect of total phenolic extract (TPE) from the stamen on the transepithelial electrical resistant (TEER) value (**A**), tight junction protein expression (**B**), and β-actin and ZO-1 staining (**C**) of Caco-2 cells. Cells were treated with 2.0 mM H_2_O_2_ 1 h, and then treated with 1 µg/mL TPE from stamen for 4 h. H_2_O_2_ treatment: cells were treated with 2.0 mM H_2_O_2_ for 1 h firstly, then treated with a regular medium for 4 h. The con. means negtive control (no H_2_O_2_ or TPE treatment). The magnification in Figure (**C**) is 40×. Red ellipse and red arrow were used to line out the changes of structure of β-actin and ZO-1, respectively. Data are expressed as mean ± SD. Different lower case letters indicate the significant difference at *p* < 0.05 for each index.

H_2_O_2_ treatment reduced TEER values (Figure 5A); however, TPE intervention restored TEER values in a significant manner (*p* < 0.05). Thus, TPE treatment reduced the damage of the Caco-2 membrane induced by H_2_O_2_. The effects of TPE from the stamen of tree peony on mRNA abundance of tight junction proteins were investigated following H_2_O_2_ pre-treatment. H_2_O_2_ treatment did not affect ZO-1 and CLDN1 mRNA abundance, but it induced an increase in CLDN3 and occludin mRNA levels. TPE intervention for 4 h further increased ZO-1, CLDN3, and occludin mRNA expression (Figure 5B). The morphology of the cytoskeleton and tight junction was visualized by actin and ZO-1 immunofluorescent staining. As shown in Figure 5C, the cytoskeletal structure of the β-actin fiber was partially disorganized by treatment with H_2_O_2_ compared with the control, while incubation with TPE after H_2_O_2_ treatment alleviated the damage induced by H_2_O_2_. The morphology of ZO-1 showed changes similar to the cytoskeleton following different treatments.

## 4. Discussion

The TPEs of the four organs showed similar phenolic profiles (Figure 1). In total, twenty-one phenolic compounds were identified and quantified (Table 1 and Table 2). Contrary to the TFC obtained using colorimetric methods, the contents of individual flavonoid glycosides determined using HPLC and the sum values of TPE from the ovary and calyx are far lower than those of the petal and stamen. This result is likely attributed to more protein and sugar in the ovary and calyx. High levels of identifiable flavonoid glycosides were found in the petal and stamen. Li et al. [8] identified and quantified 26 flavonoid glycosides in the petals of six yellow tree peony flower cultivars. In this study, eriodictyol-*O*-glucoside, kaempferol-3,7-di-*O*-glucoside, isorhamnetin-3,7-di-*O*-glucoside, isosalipurposide, luteolin-7-*O*-glucoside, apigenin-7-*O*-glucoside, apigenin-7-*O*-neohesperidoside, and kaempferol-3-*O*-glucoside were identified and quantified in the petal, stamen, ovary, and calyx of FDB flower; however, no isosalipurposide was detected in ovary and calyx. Isosalipurposide was found at a relatively higher level in the stamen, which in turn was reported to have very high antioxidant activity owing to the hydroxyl group on position 4 of the B ring of this chalcone derivative [8].

Given the only approved edible tree peony flower cultivar, all four of the organs of the FDB flower exhibited high ORAC, DPPH, and ABTS^•+^ radical scavenging activities, which can be attributed to their phenolic compounds. Both the ovary and calyx presented higher TPC and TFC (Figure 2) than other organs, while the ovary gave the highest DPPH and ABTS^•+^ radical scavenging activities (Table 3). These findings suggest that these two—often overlooked—organs could also be used as antioxidant food ingredients. In this study, the petal had the highest ORAC values, which were not consistent with values obtained using the DPPH and ABTS^•+^ assays (Table 3). The electron transfer mechanism prevails in the latter, while ORAC is based on the hydrogen–atom transfer mechanism [28].

Usually, the occurrence of intestinal diseases is associated with a defective barrier function caused by the injury from oxidation [29]. Hence, preventing oxidative damage and repairing the intestinal barrier should be effective to prevent intestinal diseases occurrence. It has been proven that phytochemicals could reduce the production of cellular ROS. In the present study, an oxidative damage model of Caco-2 cells was established using H_2_O_2_ induction, aiming to evaluate the therapeutic effects of TPE on intestinal oxidative damage. Interestingly, at a low concentration of H_2_O_2_ (0.8 mM), TPE addition increased the viability (Figure 3B,C). However, only H_2_O_2_ (0.8 mM) and TPE (from 0 to 100 μg/mL) treatment showed no impact on cell viability (Figure S1), indicating that the reaction between H_2_O_2_ and TPE might produce some unknown components with antioxidant activity or could stimulate cell metabolism. A higher concentration of H_2_O_2_ (2.0 mM) contributed to the death of cells (Figure 3A) and the positive effect of TPE could no longer be displayed (Figure 3B). Cell viability was increased when the cells were washed with PBS after treatment with 2.0 mM of H_2_O_2_ and then treatment with TPE (Figure 3C). The effect of TPE from the petal, stamen, calyx, and ovary of the tree peony flower on cell viability after H_2_O_2_ treatment is depicted in Figure 3D. TPE from the stamen was more effective for recovering the cell viability compared with that from the other three organs. However, TPC (Figure 2) and free radical scavenging ability (Table 3) of the stamen were relatively low, likely owing to the method used for presenting the data. The total phenol content and free radical scavenging ability were calculated by the dry weight of the flower, while the cell study was conducted with a consistent concentration of TPE (1.0 μg/mL). These findings also indicated that TPE from the stamen likely contains more components that assisted in combating oxidative damage. Compared with the other three organs, phenolics identified from the stamen included a high content of methyl digallate (8.05% of total), penta-*O*-galloyl-glucose (15.38% of total identified phenolics), and a relatively higher level of isosalipurposide (Table 2). Hence, it was demonstrated that these three phenolic components might be responsible for the relatively high antioxidant activity.

H_2_O_2_-induced oxidative stress triggered an imbalanced redox state and excessive ROS accumulation in Caco-2 cells, indicating that H_2_O_2_ treatment induced oxidative damage to the cells. However, a follow-up TPE treatment for 4 h significantly decreased ROS level (Figure 4A), indicating that TPE from the stamen of the tree peony flower had a high ability for ROS scavenging (Table 3). GSH is an important component of the biological redox system; therefore, its total content and the ratio of reduced GSH to GSSG can partially reveal the status of redox. H_2_O_2_ treatment decreased total GSH content, but did not affect GSSG content (Figure 4B), indicating that H_2_O_2_ treatment initiated the consumption of reduced GSH. However, follow-up TPE treatment decreased GSSG content, although the total GSH content was unaltered. Hence, the ratio of reduced GSH to GSSG increased. This was evidence of TPE playing an important role in the direct scavenging of ROS, causing decreased consumption of reduced GSH (Figure 4C). In addition, TPE treatment up-regulated the mRNA abundance of GPX-Px and SOD (Figure 4D), which are the critical antioxidant enzymes [30]. However, HO-1 expression was not affected. This observation illustrated that TPE did not stimulate the Nrf-2 pathway in which HO-1 expression acts as the downstream signal transmitting messenger [31].

The intact gut barrier is essential to prevent cell damage and restore cellular function caused by intestinal oxidative stress [32,33,34,35]. The TEER value is an important indicator to evaluate the integrity and tightness of the barrier formed by epithelial cells [36]. The barrier function of Caco-2 cells was measured until there was the formation of the stable layer. The H_2_O_2_ treatment lowered the TEER value, suggesting an increase in cell permeability [37], and a defective barrier function of cells. TEER values increased after 4 h intervention with TPE, indicating that the barrier function of Caco-2 cells was significantly enhanced. It was shown that phytochemicals from apples could enhance the TEER value of Caco-2 cells [38], and this could potentially be attributed to the antioxidant effects of TPE from the tree peony flower. In the present study, the mRNA level of tight junction proteins including ZO-1, CLDN1, CLDN3, and occludin was determined (Figure 5B). H_2_O_2_ treatment increased the mRNA expression of CLDN3 and occludin. When compared with the sole H_2_O_2_ treatment, TPE enhanced the mRNA abundance of ZO-1, CLDN3, and occludin, but did not affect the CLDN1 mRNA level in the Caco-2 cells. This could be attributed to the different sensitivity of gene expression to H_2_O_2_ [39]. In addition, the morphology of the cytoskeleton and tight junction was visualized by β-actin and ZO-1 immunofluorescent staining. H_2_O_2_ treatment disorganized the β-actin fibrin and diffused ZO-1, indicating that H_2_O_2_ caused oxidative damage to the cell membrane monolayer. The addition of TPE obviously repaired cell structure and stabilized morphological characteristics of ZO-1 (Figure 5C). These findings indicated that TPE could regulate the tight junction protein mRNA expression and repair the structure of ZO-1 protein to enhance the barrier function of Caco-2 cells.

## 5. Conclusions

In conclusion, all four of the organs of the FDB flowers displayed an abundance of phenolic compounds including flavonoid glycosides, which displayed high antioxidant activities. TPE from the FDB flowers enhanced cell viability because of its direct antioxidative effect, regulation of antioxidant enzyme expression, and enhancement of the barrier function of Caco-2 cells. It was suggested that each organ of the FDB flower or the whole flower could be used as a functional food material.

## Figures and Tables

**Table 1:** Individual phenolic compounds identified in the methanol extracts of the edible tree peony flower by HPLC-quadrupole time-of-flight mass spectrometer (Q-TOF-MS) ^a^.

No.	Retention Time	(M – H)^−^ (*m*/*z*)	UV λmax (nm)	Formula	*m*/*z* of Main Fragments (Relative Intensity, %), MS/MS	Compound Identified
1	3.57	331	278	C_13_H_16_O_10_	271(18), 211(18), 169(45), 125(15)	galloyl hexose
2	5.18	169	270	C_7_H_6_O_5_	125	gallic acid ^a^
3	8.70	299	260	C_13_H_16_O_8_	239(40), 209(15), 179(75), 137(100), 119(27)	*p*-hydroxybenzoyl hexose
4	15.13	183	270	C_8_H_8_O_5_	168(25), 124(100)	methyl gallate ^a^
5	24.4	449	285/330	C_21_H_22_O_11_	421(20), 287(100), 259(88), 243(8), 215(12), 179(10), 151(10), 125(23)	eriodictyol-*O*- glucoside
6	37.73	609	265/345	C_27_H_30_O_16_	447(60), 327(8), 285(70), 283(72), 255(10)	kaempferol-3,7-di-*O*-glucoside
7	39.85	639	254/352	C_28_H_32_O_17_	477(60), 519(5), 315(70), 313(66), 300(14)	isorhamnetin-3,7-di-*O*-glucoside
8	41.30	335	278	C_15_H_12_O_9_	183(100), 168(8), 124(40)	methyl digallate
9	42.60	335	278	C_15_H_12_O_9_	183(100), 168(8), 124(40)	methyl digallate
10	43.65	335	278	C_15_H_12_O_9_	183(100), 168(8), 124(40)	methyl digallate
11	44.78	335	278	C_15_H_12_O_9_	183(100), 168(8), 124(40)	methyl digallate
12	45.50	939	274/359	C_41_H_32_O_26_	787(6), 769(30), 617(12), 601(5), 465(6), 447(6), 431(6), 295(6), 277(6), 169(20)	penta-*O*-galloyl-glucose
13	46.06	433	267/348	C_21_H_22_O_10_	271(100), 177(5), 151(28), 119(13)	isosalipurposide
14	46.68	1091	280	C_48_H_34_O_30_	469(100), 393(6), 317(4), 295(5), 241(6), 169(65), 125(30)	hexa-*O*-galloyl-glucose
15	47.43	1091	280	C_48_H_34_O_30_	939(100), 787(8), 769(40), 617(10), 599(4), 447(6), 431(4), 169(5)	hexa-*O*-galloyl-glucose
16	47.77	1091	280	C_48_H_34_O_30_	939(100), 787(8), 769(40), 617(10), 599(4), 447(6), 431(4), 169(5)	hexa-*O*-galloyl-glucose
17	48.43	1243	279	C_55_H_36_O_34_	1091(48), 939(100), 787(4), 769(40), 617(2), 599(3), 447(3), 431(1), 169(1)	hepta-*O*-galloyl-glucose
18	48.85	447	266/365	C_21_H_20_O_11_	285(65), 284(40), 257(20), 151(40)	luteolin-7-*O*-glucoside
19	49.63	431	267/340	C_21_H_20_O_10_	268(100), 269(40)	apigenin-7-*O*-glucoside
20	50.22	577	267/340	C_27_H_30_O_14_	269(100)	apigenin-7-*O*-neohesperidoside
21	50.63	447	265/346	C_21_H_20_O_11_	285(30), 284(52), 255(66), 227(52)	kaempferol-3-*O*-glucoside

^a^ Identification of the compounds was confirmed by the authentic standard. All other compounds were tentatively identified by comparing their UV and mass spectral characteristics with those reported in the literature.

**Table 2:** Content of individual phenolic compounds (mg/100 g DW ^1^) in the different organs of the tree peony flower ^2^.

Compound Identified	Petal	Stamen	Calyx	Ovary
galloyl hexose	292.07 ± 15.75 ^b^	101.23 ± 7.22 ^c^	323.36 ± 2.18 ^a^	315.20 ± 2.70 ^a^
gallic acid ^a^	48.34 ± 5.89 ^b^	53.11 ± 1.11 ^ab^	59.89 ± 4.11 ^a^	38.96 ± 1.97 ^c^
*p*-hydroxybenzoyl hexose	39.98 ± 1.51 ^a^	17.52 ± 0.96 ^b^	4.59 ± 0.17 ^c^	3.42 ± 0.11 ^c^
methyl gallate ^a^	250.80 ± 12.37 ^c^	423.47 ± 18.54 ^a^	240.54 ± 3.39 ^c^	326.00 ± 3.55 ^b^
methyl digallate	21.67 ± 1.26 ^a^	14.41 ± 0.34 ^b^	22.83 ± 3.38 ^a^	14.34 ± 0.30 ^b^
methyl digallate	20.01 ± 1.33 ^a^	12.60 ± 0.33 ^b^	n.d.	n.d.
methyl digallate	12.37 ± 0.34 ^b^	19.18 ± 0.94 ^a^	19.02 ± 1.67 ^a^	12.80 ± 0.12 ^b^
methyl digallate	53.95 ± 8.89 ^b^	222.40 ± 8.14 ^a^	53.57 ± 6.13 ^b^	47.89 ± 8.66 ^b^
penta-O-galloyl-glucose	413.82 ± 9.83 ^b^	513.10 ± 31.68 ^a^	246.10 ± 1.38 ^c^	265.78 ± 1.03 ^c^
hexa-O-galloyl-glucose	328.36 ± 18.54 ^a^	198.72 ± 2.97 ^b^	308.29 ± 1.16 ^a^	329.73 ± 1.86 ^a^
hexa-O-galloyl-glucose	52.99 ± 2.46 ^c^	6.88 ± 0.11 ^d^	141.71 ± 3.64 ^a^	91.86 ± 2.42 ^b^
hexa-O-galloyl-glucose	91.80 ± 3.33 ^a^	62.28 ± 1.12 ^b^	19.59 ± 0.41 ^c^	47.39 ± 1.21 ^b^
hepta-O-galloyl-glucose	246.10 ± 17.91 ^b^	52.37 ± 3.88 ^d^	207.68 ± 9.27 ^c^	281.72 ± 4.87 ^a^
Total identified phenolic acids content	1872.26 ± 103.03 ^a^	1697.26 ± 31.40 ^b^	1647.18 ± 12.53 ^b^	1775.09 ± 11.36 ^ab^
eriodictyol-*O*- glucoside	45.73 ± 2.96 ^a^	16.35 ± 0.38 ^b^	1.34 ± 0.02 ^c^	0.99 ± 0.01 ^c^
kaempferol- 3,7-di-O-glucoside	1124.33 ± 41.99 ^a^	601.44 ± 13.24 ^b^	91.47 ± 2.93 ^c^	27.75 ± 0.61 ^d^
isorhamnetin -3,7-di-O-glucoside	11.58 ± 0.70 ^d^	56.35 ± 1.22 ^b^	74.82 ± 0.19 ^a^	18.03 ± 0.16 ^c^
isosalipurposide	3.49 ± 0.24 ^b^	33.22 ± 0.25 ^a^	n.d.	n.d.
luteolin-7-O-glucoside	155.43 ± 9.84 ^a^	116.35 ± 2.11 ^b^	10.23 ± 0.29 ^c^	5.51 ± 0.10 ^c^
apigenin-7-O-glucoside	475.08 ± 45.41 ^a^	414.69 ± 14.82 ^a^	115.13 ± 3.26 ^b^	31.41 ± 1.05 ^c^
apigenin-7-O- neohesperidoside	518.95 ± 5.37 ^a^	372.05 ± 12.05 ^b^	124.07 ± 2.99 ^c^	31.90 ± 0.34 ^d^
kaempferol-3-O-glucoside	27.27 ± 2.17 ^a^	27.63 ± 1.55 ^a^	21.18 ± 1.75 ^b^	10.64 ± 0.32 ^c^
Total identified flavonoids content	2361.87 ± 17.87 ^a^	1638.07 ± 42.54 ^b^	438.64 ± 11.34 ^c^	126.23 ± 7.18 ^d^
Total identified phenolics content	4234.12 ± 120.90 ^a^	3335.33 ± 45.20 ^b^	2085.83 ± 14.79 ^c^	1901.32 ± 11.67 ^d^

^1^ DW, dry weight of sample. ^2^ Results are expressed as mean ± SD. Values with no letters in common are significantly different (*p* < 0.05). n.d., not detected.

**Table 3:** Oxygen radical absorbance capacity (ORAC), 2,2′-azino-bis (3-ethylbenzothiazoline-6-sulfonic acid) diammonium salt (ABTS^•+^), and 2,2′-diphenyl-1-picrylhydrazyl (DPPH) radical scavenging activity of the methanol extracts from different organs of the tree peony flower.

Samples	DPPH (μmol trolox/g DW)	ABTS^•+^ (μmol trolox/g DW)	ORAC (μmol trolox/g DW)
Petal	325.69 ± 9.99 ^b^	345.08 ± 14.18 ^c^	1061.03 ± 55.06 ^a^
Stamen	207.80 ± 7.73 ^c^	234.58 ± 7.67 ^d^	704.06 ± 91.57 ^c^
Calyx	433.57 ± 4.60 ^a^	582.33 ± 10.36 ^b^	828.84 ± 20.97 ^b^
Ovary	444.58 ± 13.84 ^a^	610.21 ± 24.33 ^a^	555.11 ± 43.23 ^d^

Results are expressed as mean ± SD. Values with no letters in common are significantly different (*p* < 0.05) for each index. DW, dry weight of sample.

## Supplementary Materials

The following are available online at https://www.mdpi.com/2304-8158/8/10/471/s1, Figure S1: Effect of H_2_O_2_ and TPE on Caco-2 cell viability, Table S1: Primers used in this study.
