# Supplementary material for: Phenolic Profile and Antioxidant Activity of the Edible Tree Peony Flower and Underlying Mechanisms of Preventive Effect on H2O2-Induced Oxidative Damage in Caco-2 Cells
Source: Foods. 2019 Oct 10;8(10):471. doi: 10.3390/foods8100471 (PMC6835411; doi:10.3390/foods8100471)
Supplement: Supplementary file 1 [file foods-08-00471-s001.pdf]

Supplementary Data for

Phenolic profile and antioxidant activity of the edible tree peony flower and underlying mechanisms of preventive effect on H_2O_2-induced oxidative damage in Caco-2 cells

Primers detailed information

Table S1 Primers used in this study

Gene	Primer sequences (5′ →3′)	Length (bp)	Access No.
ZO-1	CAACATACAGTGACGCTTCACA<br>CACTATTGACGTTTCCCCACTC	105	NM_001355015.1
Occludin	GACTATGTGGAAAGAGTTGAC<br>ACCGCTGCTGTAACGAG	174	XM_017008914.2
CLDN1	TGGTGGTTGGCATCCTCCTG<br>AATTCGTACCTGGCATTGACTGG	232	NM_021101.4
CLDN3	GCCACCAAGGTCGTCTACTC<br>CCTGCGTCTGTCCCTTAGAC	101	NM_001306.3
SOD	ATCCTCTATCCAGAAAACACG<br>ACACCACAAGCCAAACGAC	250	NM_000454.4
HO-1	TTTGAGGAGTTGCAGGAGC<br>GTAAGGACCCATCGGAGAA	179	NM_002133.2
GSH-Px	CCTCTAAACCTACGAGGGAGGAA<br>GGGAAACTCGCCTTGGTCT	107	NM_001329455.1
β-Actin	CACCAACTGGGACGACAT<br>ACAGCCTGGATAGCAACG	189	NM_001101.5

Effect of H_2O_2 and TPE on Caco-2 cell viability

As shown in Fig. 4, the viability of Caco-2 cell did not change when H_2O_2 concentration was from 0 to 0.8 mM. However, it decreased significantly when H_2O_2 concentration increased from 1.0 to 2.0 mM and showed insignificance between 2.0 and 2.5 mM of H_2O_2 concentration (Fig. S1A). TPE of 0-100 ug/mL was selected to test its effect on Caco-2 cell viability. It was found that the concentration had no significant effect on cell activity (Fig. S1B).

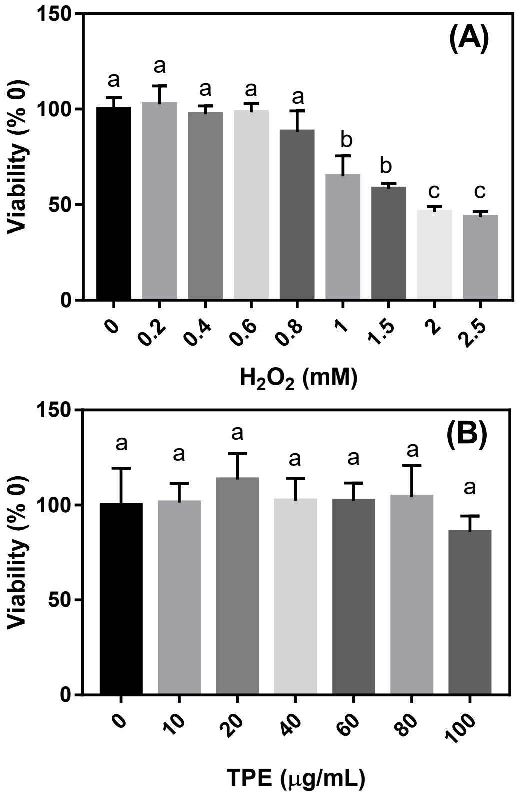

Fig. S1 Effect of H_2O_2 and TPE on Caco-2 cell viability. Data are expressed as mean±SD. Different lower case letters indicate the significant difference at $P<0.05$.

Identification of phenolic compounds by HPLC-DAD-ESI-MS^2

Phenolic acids and gallotannins

As shown in Table 1, compound 1, which showed a $[M-H]^-$ ion at m/z 331 and yielded the main fragmention at m/z 169 corresponding to the loss of a hexose, was identified as galloyl hexose. The molecular ion of compound 2 was at m/z 169, with characteristic MS^2 fragment ions at m/z 125 corresponding to a loss of 44 Da resulting from the elimination of a CO_2 molecular species. Compound 2 was positively identified as gallic acid using the same standard.

Compound 3 was characterized as a hydroxybenzoic acid glycoside at m/z 299 with a six-carbon sugar esterified to its carboxylic acid. Secondary ionization produced fragments (m/z 239, 209, and 179) typical of an acid-resistant glycosidic bond corresponding to the ring fission of glucose that produced 60, 90, and 120 Da neutral fragments as previously reported for C-linked glycosides (Filip & Magda, 2004). The corresponding aglycone at m/z 137 corresponded to a *p*-hydroxybenzoic acid on the basis of its characteristic UV spectral as compared to a standard of *p*-hydroxybenzoic acid. Compound 3 was identified as p-hydroxybenzoyl hexose according to the literature (Fan, Zhu, Kang, Ma, & Tao, 2012). The deprotonated molecular ion of compound 4 was at m/z 183, with characteristic MS^2 fragment ions at m/z 168 and m/z 124. The former resulted from the loss of methyl radical, and the latter was due to further elimination of a CO_2 molecule. Compound 4 was unambiguously identified as methyl gallate using the same standard.

The UV spectra of compounds 8-11 were suggestive of gallate-type compounds, although the λ max at 278 nm was quite broad. The same molecular ion $[M-H]^-$ at m/z 335 was presented, with major fragment ions at m/z 183, m/z 168 and m/z 124, suggesting the presence of a methyl gallate moiety. Compounds 8-11 were identified as methyl digallate isomers according to the literature (Barreto, et al., 2008).

The typical UV spectrum of galloyl-glucoses (GGs) has two maxima at 218 and 280 nm (Salminen, Ossipov, Loponen, Haukioja, & Pihlaja, 1999). All GGs and gallotannins elute in the order of increasing degree of galloylation (Salminen, et al., 1999). When GGs contain more than five galloyl groups, they are called gallotannins. Gallotannins have a characteristic shoulder in their UV spectra at around 300 nm due to the meta-depsidic digalloyl groups present in the structure (Arapitsas, Menichetti, Vincieri, & Romani, 2007). Compounds 12, 14-17 showed nearly identical UV spectra

which were very similar to that of gallic acid. Compound 12 exhibited a [M-H]$^-$ ion at m/z 939 and fragment ions at m/z 787 and 769 caused by the loss of galloyl (152 Da) and gallic acid (170 Da) moieties, respectively. Further loss of galloyl and gallic acid moieties revealed a fragmentation pattern typical of *penta-O*-galloyl-glucoside (Barreto, et al., 2008). Compound 14-16 exhibited a [M-H]$^-$ ion at m/z 1091 and fragment ions at m/z 939 and 787 caused by the loss of two successive galloyl (152 Da) moieties, respectively. Further loss of galloyl and gallic acid moieties revealed a fragmentation pattern typical of *hexa-O*-galloyl-glucoside (Barreto, et al., 2008; Nicolai, Reinhold, & Andreas, 2004). Compound 17 presented a [M-H]$^-$ ion at m/z 1243 and fragment ions at m/z 1091 and 939 caused by the loss of two successive galloyl (152 Da) moieties, respectively. Further loss of galloyl and gallic acid moieties revealed a fragmentation pattern typical of *hepta-O*-galloyl-glucoside.

Flavonoids

Flavonoids comprise another family of phenolic compounds present in peony flower. Eight flavonoid glycosides were tentatively identified including peaks 5, 6, 7, 13, 18, 19, 20, and 21, which exhibited the typical UV spectra of flavonoid (Table 1).

Compound 5 presented [M-H]$^-$ at m/z 449 and a major MS/MS fragment of m/z 287 [M-H-162]$^-$ accounted for the loss of a hexose. Compound 5 was identified as eriodictyol-O-glucoside, which had been reported in *Moraceae* family (Ammar, Contreras, Belguith-Hadrich, Bouaziz, & Segura-Carretero, 2015). This appears to be the first report of the presence of eriodictyol-O-hexoside in peony flower. Compound 6 presented [M-H]$^-$ at m/z 609, MS/MS fragments of m/z 447 [M-H-162]$^-$ and 285 [M-H-162-162]$^-$ accounted for the successive loss of a hexose. It was identified as kaempferol-3,7-di-O-glucoside, which was previously separated and identified in the petal from *Paeonia rockii* (Zhang, et al., 2016). Peak 7 which presented [M-H]$^-$ at m/z 639 and was identified as isorhamnetin-3,7-di-O-glucoside, had fragments of m/z 477 [M-H-162]$^-$ and 315 [M-H-162-162]$^-$ which was in agreement with a previous report (Li, Kuang, Chen, & Zeng, 2016). Compound 13, which presented [M-H]$^-$ at m/z 433, with a major fragmentation at m/z 271 corresponding to the loss of a hexose, was identified as isosalipurposide (Li, et al., 2009).

Compound 18 which presented UV spectrum at 266/365 and [M-H]$^-$ at m/z 447, with a major fragmentation at m/z 285 corresponding to the loss of hexose, was identified as luteolin-7-O-glucoside, which was also identified in *Paeonia rockii* using authentic standard by Li, et al. (2016). Compound 19 which exhibited [M-H]$^-$ at m/z 431 and fragmentations at m/z 269 [M-H-162]$^-$ and 268 [M-H-1-162]$^-$

corresponding to the loss of glucosyl, was identified as apigenin-7-O-glucoside which has been separated and identified in the petal from *Paeonia rockii* (Zhang, et al., 2016). Compound 20 exhibited $[M-H]^-$ at m/z 577, and fragmentations at m/z 431 $[M-H-146]^-$ and 269 $[M-H-146-162]^-$ corresponding to the loss of rhamnosyl and glucosyl, respectively. Compound 20 was identified as apigenin 7-O-neohesperidoside (Li, et al., 2009). Compound 21 presented UV spectrum at 265/346 and $[M-H]^-$ at m/z 447, with co-existence of MS/MS fragments at m/z 285 and 284 suggesting the attachment of glycoside to the 3-OH (Zhang, et al., 2019). It was identified as kaempferol-3-O-glucoside (Granica, Kluge, Horn, Matkowski, & Kiss, 2015).

Literatures:

Ammar, S., Contreras, M. d. M., Belguith-Hadrich, O., Bouaziz, M., & Segura-Carretero, A. (2015). New insights into the qualitative phenolic profile of *Ficus carica* L. fruits and leaves from Tunisia using ultra-high-performance liquid chromatography coupled to quadrupole-time-of-flight mass spectrometry and their antioxidant activity. *RSC Advances, 5*(26), 20035-20050.

Arapitsas, P., Menichetti, S., Vincieri, F. F., & Romani, A. (2007). Hydrolyzable tannins with the hexahydroxydiphenoyl unit and the m-Depsidic link: HPLC-DAD-MS identification and model synthesis. *Journal of Agricultural and Food Chemistry, 55*(1), 48-55.

Barreto, J. C., Trevisan, M. T. S., Hull, W. E., Erben, G., de Brito, E. S., Pfundstein, B., et al. (2008). Characterization and quantitation of polyphenolic compounds in bark, kernel, leaves, and peel of mango (*Mangifera indica* L.). *Journal of Agricultural and Food Chemistry, 56*(14), 5599-5610.

Fan, J., Zhu, W., Kang, H., Ma, H., & Tao, G. (2012). Flavonoid constituents and antioxidant capacity in flowers of different Zhongyuan tree penoy cultivars. *Journal of Functional Foods, 4*(1), 147-157.

Filip, C., & Magda, C. (2004). Mass spectrometry in the structural analysis of flavonoids. *Journal of Mass Spectrometry, 39*(1), 1-15.

Granica, S., Kluge, H., Horn, G., Matkowski, A., & Kiss, A. K. (2015). The phytochemical investigation of Agrimonia eupatoria L. and Agrimonia procera Wallr. as valid sources of Agrimoniae herba--The pharmacopoeial plant material. *J Pharm Biomed Anal, 114*, 272-279.

Li, C., Du, H., Wang, L., Shu, Q., Zheng, Y., Xu, Y., et al. (2009). Flavonoid Composition and Antioxidant Activity of Tree Peony (Paeonia Section Moutan) Yellow Flowers. *Journal of Agricultural and Food Chemistry, 57*(18), 8496-8503.

Li, J., Kuang, G., Chen, X., & Zeng, R. (2016). Identification of chemical composition of leaves and flowers from paeonia rockii by UHPLC-Q-exactive qrbitrap HRMS. *Molecules, 21*(7), 947.

Nicolai, B., Reinhold, C., & Andreas, S. (2004). Characterization of gallotannins and benzophenone derivatives from mango (Mangifera indica L. cv. 'Tommy Atkins') peels, pulp and kernels by high-performance liquid chromatography/electrospray ionization mass spectrometry. *Rapid Communications in Mass Spectrometry, 18*(19), 2208-2216.

Salminen, J. P., Ossipov, V., Loponen, J., Haukioja, E., & Pihlaja, K. (1999). Characterisation of hydrolysable tannins from leaves of *Betula pubescens* by high-performance liquid chromatography–mass spectrometry. *Journal of Chromatography A, 864*(2), 283-291.

Zhang, H., Li, X., Wu, K., Wang, M., Liu, P., Wang, X., et al. (2016). Antioxidant activities and chemical constituents of flavonoids from the flower of paeonia ostii. *Molecules, 22*(1), 5.

Zhang, L., Xu, L., Ye, Y.-h., Zhu, M.-f., Li, J., Tu, Z.-c., et al. (2019). Phytochemical profiles and screening of α-glucosidase inhibitors of four Acer species leaves with ultra-filtration combined with UPLC-QTOF-MS/MS. *Industrial Crops and Products, 129*, 156-168.
